# Maximum Efficacy of Mesenchymal Stem Cells in Rat Model of Renal Ischemia-Reperfusion Injury: Renal Artery Administration with Optimal Numbers

**DOI:** 10.1371/journal.pone.0092347

**Published:** 2014-03-17

**Authors:** Jieru Cai, Xiaofang Yu, Rende Xu, Yi Fang, Xiaoqin Qian, Shaopeng Liu, jie Teng, Xiaoqiang Ding

**Affiliations:** 1 Department of Nephrology, Zhongshan Hospital, Fudan University, Shanghai, China; 2 Department of Cardiology, Renji Hospital, Shanghai Jiaotong University, Shanghai, China; 3 Department of Ultrasonography, Zhongshan Hospital, Fudan University, Shanghai, China; 3 Blood Purification Center, Zhongshan Hospital, Fudan University, Shanghai, China; Children’s Hospital Boston/Harvard Medical School, United States of America

## Abstract

**Backgrounds:**

Despite the potential therapeutic benefits, cell therapy in renal ischemia-reperfusion (I/R) injury is currently limited by low rates of cell engraftment after systemic delivery. In this study, we investigate whether locally administration through renal artery can enhance the migration and therapeutic potential of mesenchymal stem cells (MSCs) in ischemic kidney.

**Methods:**

The model of renal I/R injury was induced by 45 min occlusion of the left renal pedicle and right nephrectomy in rat. Followed by reperfusion, graded doses of CM-Dil labeled MSCs were implanted via three routes: tail vein (TV), carotid artery (CA), and renal artery (RA). Renal blood flow was evaluated by color and spectral Doppler ultrasound at 1 h and 24 h post-I/R. All the samples were collected for analysis at 24 h post-I/R.

**Results:**

After injection of 1×10^6^ MSCs, RA group showed obviously increased renal retention of grafted MSCs compared with TV and CA group; however, the renal function was even further deteriorated. When graded doses of MSCs, the maximal therapeutic efficiency was achieved with renal artery injection of 1×10^5^ MSCs, which was significantly better than TV and CA group of 1×10^6 ^MSCs. In addition, further fluorescent microscopic and ultrasonic examination confirmed that the aggravated renal dysfunction in RA group was due to renal hypoperfusion caused by cell occlusion.

**Conclusion:**

Administration route and dosage are two critical factors determining the efficiency of cell therapy and 1×10^5^ MSCs injected through renal artery produces the most dramatic improvement in renal function and morphology in rat model of renal I/R injury.

## Introduction

Acute kidney injury (AKI) is a complex clinical syndrome with a high incidence of 3%–5% in general hospital, characterized by acute tubular injury and rapid renal dysfunction, generally caused by ischemic or toxic insults [1]–[3]. The kidney undergoing I/R results in extensive and complex inflammatory/oxidative stress responses [4], [5]. Although many efforts have been made to deal with this problem, such as new drugs and modern dialysis techniques, innovative interventions beyond supportive therapy are not yet available [3], [6]. Thus, it is urgent for us to explore novel therapeutic strategies to attenuate AKI or expedite recovery.

Relevant researches have demonstrated that cell therapy is a promising alternative for the treatment of AKI, and several studies have suggested that administration of mesenchymal stem cells (MSCs) may protect AKI experimental model from ischemia-reperfusion (I/R) injury. Although the mechanism for the effects remains controversial, many studies have found that their early protection is primarily mediated by complex paracrine actions, such as immunomodulation and growth factor secretion [7]–[11]. Thus, effective and targeted delivery of cells to injured organs is critical determinants in the success of cell therapy [12].

At present, there are no unified delivery routes and dosage for cell therapy in animal models of AKI. Systemic delivery (intravenous and intra-arterial injections) is the most commonly used route for MSCs administration. However, after systemic administration, the grafted MSCs appear to be trapped in tissues rich with capillaries, such as lung, or rapidly lost a high proportion of cells into the systemic circulation within a few minutes [13], [14]. Although directive local injection (intraparenchymal injection) may increase the retention and survival of administrated MSCs to some extent, this approach also results in regional storage of MSCs [15]. Based on these conditions, we considered an alternative route that local transplantation of MSCs through renal artery directly. Theoretically, administration MSCs through renal artery can increase the retention of donor cells and enables them homogeneous dissemination to the damaged kidney in a more physiological manner. In this study, we compared the therapeutic efficacy of MSCs with different administration routes and dosages in the treatment of ischemic kidney injury.

## Materials and Methods

### Animals

Male Sprague-Dawley (SD) rats weighing 250–300 g were purchased from the Animal Center, Shanghai Medical College, Fudan University (Shanghai, China) and housed in a pathogen-free environment with a12 h–12 h light-dark cycle. Food and water were available ad libitum. All the experimental procedures were approved by the Animal Care and Use Committee of Zhongshan Hospital and conducted under the guidelines for Animal Care and Use of Fudan University, China.

### Isolation and Culture of Bone-marrow-derived MSCs

MSCs were isolated from Sprague-Dawley rats, weighted 50–100 g, as previously reported [16]. Cells of passages 6–8 were used for these experiments. At 80% confluence, cells were detached, and labeled with CellTracker CM-Dil (Invitrogen, USA), according to the manufacturer’s instructions. The labeling efficiency was >98%, as determined by fluorescent microscopy. MSCs were kept on ice in PBS until infusion.

### Ischemia-reperfusion Injury Rat Model, Cell Transplantation

Rats were anesthetized with intraperitoneal pentobarbital sodium (40 mg/kg). Through a midline abdominal incision, I/R injury were induced by 45 min occlusion of the left renal pedicle with nontraumatic microvascular after right nephrectomy was performed. Thereafter, the clamps were removed and kidney reperfusion was visually confirmed, and then rats were randomly allocated to control group (n = 6) and intervention group (n = 60). Immediately after reperfusion, the animals in intervention group were further divided into three groups according to the delivery routes of CM-Dil labeled MSCs administration: tail veil (TV), carotid artery (CA), and renal artery (RA). We compared the efficiency of graded number of MSCs through three routes in renal I/R injury and the subgroups were followed (n = 6 each subgroup): (1) TV group: 1×10^6^ MSCs; (2) CA group: 1×10^6^, 5×10^5^, 1×10^5^, and 5×10^4 ^MSCs; (3) RA group: 1×10^6^, 5×10^5^, 1×10^5^, 5×10^4^ MSCs, and PBS (0 MSCs). The cells were suspended in 500 μl PBS. Twenty-four hours after ischemia, the animals were reanesthetized with pentobarbital sodium, and the specimens were collected and preserved according to the standard protocol.

**Figure 1:**
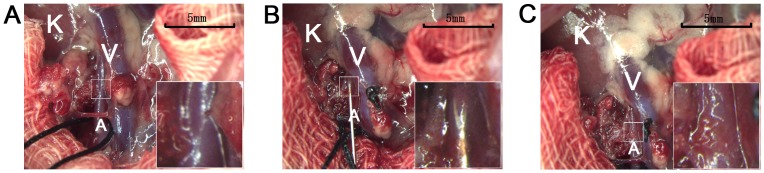
Schematic diagram of sub-adventitial renal artery puncture. (A) Find the renal artery and carefully dissected from surrounding structures under microscope. (B) Sub-adventitial renal artery puncture with a 33-gauge needle and inject the cell solution into the artery slowly. (C) Pull out the needle quickly and put the gelatin sponge over the puncture hole, then take all-purpose sponges. Abbreviation: K, the left kidney; A, the renal artery; V, the renal vein.

Without affecting the renal perfusion, those animals in the RA group received MSCs via the renal artery with a 33-gauge needle (Cadence, USA). The steps of this process were described [Fig. 1]. (1) Separate the renal artery from surrounding structure under the microscope. (2) Sub-adventitial renal artery puncture: 33-gauge needle was used to puncture into the sub-adventitial level of renal artery at the proximal segment with the help of the micro-forceps. After microscopic view confirmed needle-tip entry into the sub-adventitia, the needle was punctured through the wall of renal artery. Inject the cell solution into the artery slowly over a period of 500 μl in 3–5 minutes. (3) Pull out the needle quickly and put the gelatin sponge over the puncture hole and then take all-purpose sponges out of the abdominal cavity and close the abdomen.

### Measurement of Renal Function

Blood samples for measurement of the creatinine were collected at 24 h post-ischemia. Serum creatinine levels were determined using the Quantichrom Creatinine Assay Kit (BioAssay Systems, Hayward, CA), following the manufacturer’s protocol.

### Histological Analysis

Collected kidney samples were fixed in 10% buffered formalin, dehydrated with ethanol and embedded in paraffin. For analysis of tubular injury, 3 μm sections were stained with hematoxylin-eosin. Postischemic tubular injury was scored by assessing the percentage of tubules in the corticomedullary junction that displayed tubular cell flattening, cell necrosis, loss of brush border, and luminal cast formation, as described formerly [17]. Ten non-overlapping fields of each section were evaluated (400×) as follow: 0 = normal, 1 = 10 to 25%, 2 = 26 to 50%, 3 = 51 to 75%, and 4 = >75%.

### Assess Homing of Grafted MSCs to the Kidney and other Representative Organs

Collected samples were embedded in Tissue-Tek OCT compound (Sakura Finetechnical Co. Ltd., Japan) and preserved at −80°C. The cryostat sections of kidney tissue (10 μm) were observed using a confocal fluorescent microscope at 20× and 63× magnification (nuclei stained blue with DAPI). The cryostat sections of other organs were directly examined under fluorescent microscope (10×). CM-Dil positive cells were defined as implanted MSCs.

### Doppler Ultrasound Assessment of Renal Blood Flow

Ultrasound assessment was carried out on rats in control and RA groups at 1 h and 24 h after I/R injury. The ultrasound examinations, including color Doppler imaging and spectral Doppler analysis, were performed using a Vivid S6 ultrasound scanner (GE Medical Systems, Norway) equipped with a 12-MHz linear transducer (12 L). The findings were documented by means of the imaging technology providing comparison over time. Renal blood flow after renal artery injection was evaluated through color Doppler flow imaging and peak systolic (PS) and end-diastolic (ED) blood flow velocity in interlobar artery.

### Statistics

Statistical analyses were performed using the SPSS software (version 13.0) and presented as mean±SD. Independent *t*-test and One-way ANOVA with the LSD-*t* test were used for multiple comparisons between the groups. Statistical significance level was defined as *P*<0.05.

## Results

### The Distribution of MSCs after Infusion Through Different Administration Routes

**Figure 2:**
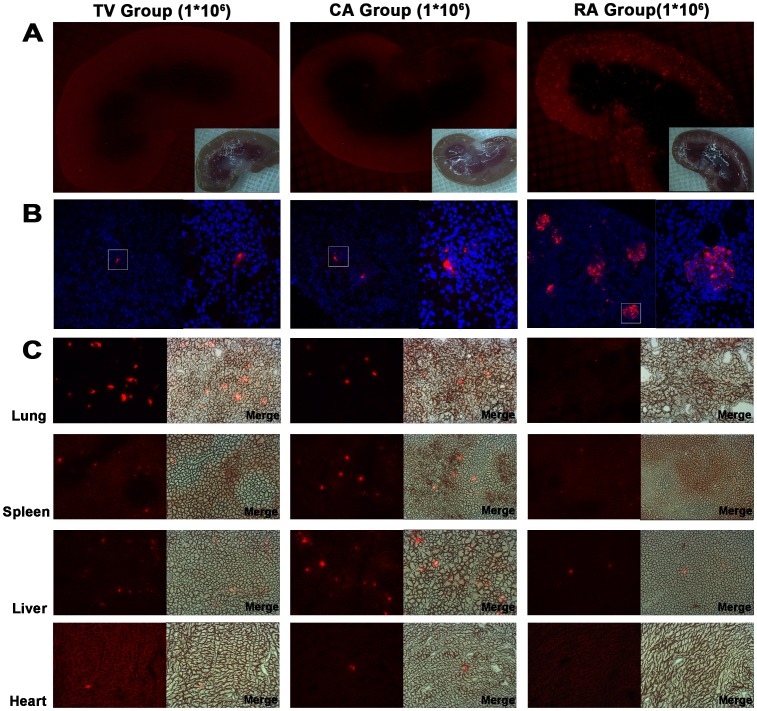
The distribution in the representative organs after infusion of 1×10^6^ MSCs through different administration routes. (A–B) Renal retention of grafted MSCs. Under fluorescence microscopic observation, both the gross specimen and frozen sections showed that there were significantly more CM-Dil labeled MSCs (red) engrafted to the kidney in RA group compared with TV and CA group. (C) The distribution of MSCs in the other representative organs, including lung, spleen, liver and heart. In TV group, the majority of implanted cells accumulated in the lung. In CA group, the transplanted cells widely distributed to these representative organs. In RA group, few cells were detected in other organs.

After administration of 1×10^6 ^MSCs through three different routes, the whole-body distribution of implanted CM-Dil labeled cells were determined under fluorescence microscopic observation of representative organs at 24 h post-I/R. First of all, we evaluated the retention of cells in the kidney. Both the gross specimen and histologic section demonstrated that obviously maximal cell retention was achieved with renal artery implantation. Compared with CA group, fewer cells delivered by means of tail vein injection were observed in the renal tissue [Fig. 2 A–B]. Next, we assessed the distribution of MSCs in the other representative organs, including heart, liver, spleen, and lung [Fig. 2 C]. In rats that received cells through tail vein, the majority of implanted cells accumulated in the lung. In CA group, along with the blood flow, these cells widely distributed to the various organs after injection. In RA group, few cells were localized to the organs other than kidney.

### The Effect of MSCs Injected via Different Routes on the Renal Function and Morphology after I/R Injury

**Figure 3:**
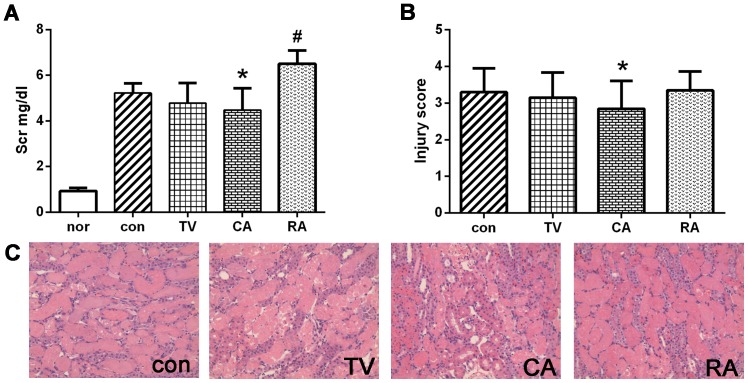
The effect of 1×10^6^ MSCs transplantation on renal function and morphology after I/R injury. (A) Compared with control group (5.22±0.43 mg/dl), both TV group (4.78±0.88 mg/dl) and CA group (4.48±0.95 mg/dl) seemed to derive at least some benefits from cell therapy, as shown by a trend towards a reduced Scr level, although the difference between TV and control group was no statistically significant. However, the Scr level further increased significantly in RA group (6.51±0.58 mg/dl). (B–C) The degree of renal injury was assessed in the juxtamedullary cortex of the sections via a semiquantitative method and representative photomicrographs were presented (HE staining, magnification 200×). When 1×10^6^ MSCs were applied, only rats in CA group showed a significant decrease in renal injury score (2.85±0.76) compared with control group (3.30±0.65, *P*<0.05). *P<0.05, *vs* control group; #*P*<0.05, *vs* control group.

Compared to normal rats, animals in the control group displayed significantly increased Scr level (5.22±0.43 mg/dl) and renal injury score (3.30±0.65) at 24 h after reperfusion [Fig. 3]. Administration of MSCs (1×10^6^ cells) through carotid artery attenuated the I/R-induced renal dysfunction, as evidenced by a decrease in Scr level (4.48±0.95 mg/dl) and renal injury score (2.85±0.76). In TV group, no statistically significant improvement was found with 1×10^6 ^MSCs injection; although there was a trend towards lower Scr level (4.78±0.88 mg/dl) and renal injury score (3.15±0.68). Interestingly, in RA group of 1×10^6 ^MSCs, cell transplantation not only failed to protect against renal I/R injury but even adversely affected renal as indicated by significantly higher Scr level (6.51±0.58 mg/dl, *P*<0.05) and injury score (3.35±0.51 mg/dl, *P*<0.05) compared to control group.

### The Safety of Renal Artery Puncture and Injection

**Figure 4:**
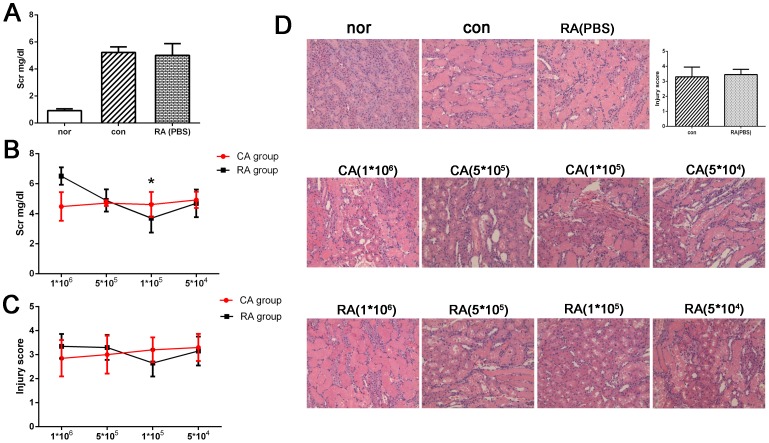
Effect of graded number of MSCs on renal function and morphology after I/R injury. (A) In RA group with PBS only, no further increasing Scr level was detected (5.01±0.87 mg/dl) compared with control group (5.22±0.43 mg/dl). (B) In CA group, the Scr level decreased with increased amounts of injected MSCs. However, in RA group, there was a U-type change and the lowest Scr level was obtained with 1×10^5 ^MSCs (3.71±0.96 mg/dl), which was significantly lower than that in all groups, including CA group and RA group (P<0.05). (C–D) Histological examination (HE staining, magnification 200×) showed that the most significant morphological improvement was found in RA group (1×10^5^), which was consistent with functional studies. *P<0.05, *vs* CA group (1×10^6^).

To determine whether the aggravated renal damage was associated with renal artery puncture and injection, we investigated the safety of the surgery. In our experiment, the bleeding from the puncture site was well controlled and no animal died during the surgery. In rats received renal injection of PBS, no further increasing of Scr level was detected at 24 h after renal I/R injury (5.01±0.87 mg/dl) compared with control group (5.22±0.43 mg/dl) [Fig. 4 A]. Moreover, histological examination revealed that there was no significant difference in the renal injury score between rats in RA group with PBS injection (3.45±0.35) and in control group (3.30±0.65) [Fig. 4 D]. These results indicated that renal artery puncture and injection would not cause any secondary damage, and was safe in rat model of renal I/R injury.

### Relation between the Amount of Injected MSCs and Therapeutic Efficacy

To further explore the apparent contradiction between increased homing of MSCs to the kidney and further deteriorated renal function in RA group, we evaluated the renal function with decreased cell delivery. Different from CA group, in which the improvement of renal function was positively correlated with injected cell dosage, rats in RA group displayed a U-type change of Scr level, with the lowest Scr level occurring when 1×10^5^ MSCs were applied [Fig. 4 B]. In addition, compared with CA group of 1×10^6 ^MSCs (4.48±0.95 mg/dl), in which the best therapeutic effect was obtained in the comparison of different routes with some amount of cells, the Scr level was further significantly lower in RA group of 1×10^5 ^MSCs (3.71±0.96 mg/dl, *P*<0.05). Histological examination confirmed that the degree of renal injury was consistent with the change of renal function [Fig. 4 C–D].

### Localization of Renal Arterially Injected MSCs and its Affection on Renal Blood Flow

**Figure 5:**
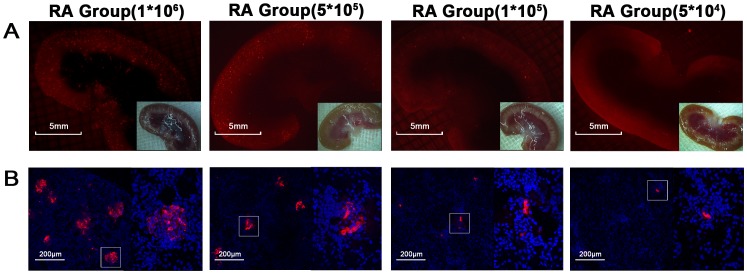
Intra-renal localization of injected MSCs in RA group. A significant trend toward increased cell retention in the kidney was noted with increased amounts of injected MSCs under fluorescent microscopic examination of both gross specimen (A) and frozen section (B), and further observation showed that most of the increased cells accumulated in the renal glomeruli.

In RA groups, CM-Dil labeled MSCs were identified within the renal cortex at 24 h post I/R, and renal retention was increased with the number of injected cells [Fig. 5 A]. However, further fluorescent microscopic observation indicated that most of the MSCs homing to the kidney were localized primarily to the renal glomeruli but not to the renal tubules [Fig. 5 B]. Especially in RA group with 5×10^5^ and 1×10^6^ cells, many cells accumulated in renal glomeruli, which may block local blood flow and therefore aggravate renal injury.

**Figure 6:**
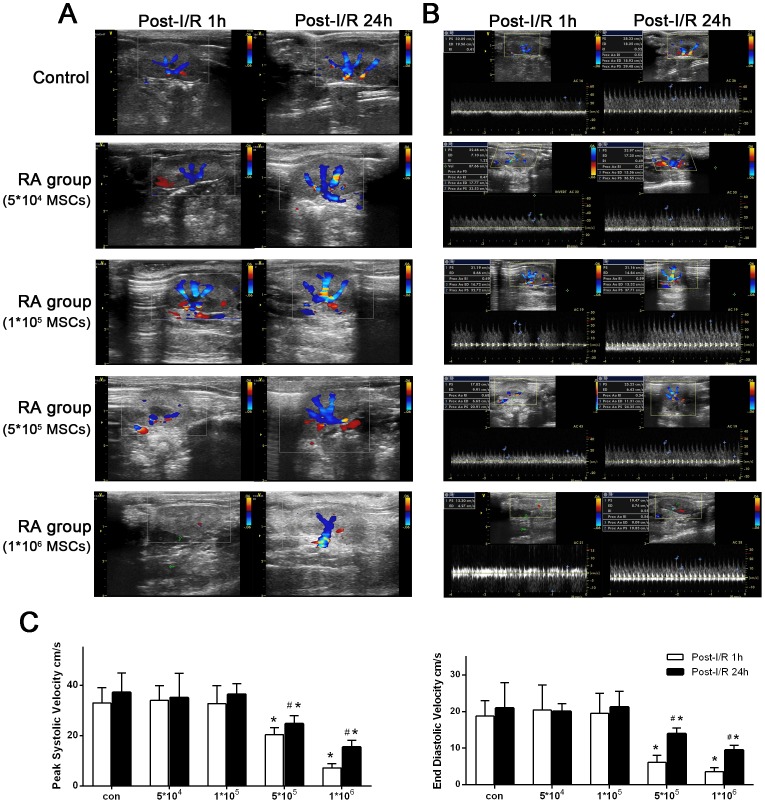
Ultrasonic assessment of the affection of renal arterially injected MSCs on renal blood flow. (A) Color Doppler imaging of renal blood flow. Compared with control group, animals in RA group of 5×10^5^ and 1×10^6^ showed significant hypoperfusion of renal parenchyma, especially at 1 h after I/R. (B–C) Spectral Doppler ultrasonic evaluation. In consistent with color Doppler results, spectral Doppler data confirmed that both PS and ED blood flow velocity dramatically failed in the RA group of 5×10^5^ and 1×10^6^ cells, which indicated poor peripheral perfusion of the kidney. *P<0.05, *vs* control group at same time, #*P*<0.05, *vs* 1 h post I/R at same group.

In order to evaluate the effect of renal arterially delivered MSCs on renal hemodynamics, color and spectral Doppler ultrasound examination of renal blood flow was conducted. The characteristics of parenchymal perfusion based on the color Doppler imaging were evaluated according to the Turetschek et al. scale [18][Fig. 6 A]. Significant hypoperfusion of parenchyma was observed in rats received 5×10^5^ and 1×10^6^ cells through renal artery at 1 h post-I/R, and these animals recovered to better parenchyma perfusion at 24 h post-I/R; while rats in control group and in the RA group with 5×10^4^ and 1×10^5^ cells showed similar color Doppler imaging of parenchyma perfusion at both 1 h and 24 h after I/R. PS and ED blood flow velocity, measured at the level of interlobar arteries by spectral Doppler ultrasound, was used for evaluation of the peripheral perfusion [Fig. 6 B–C]. Post-I/R 1 h, compared with control group, both PS and ED blood flow velocity dramatically failed in the RA group with 5×10^5^ and 1×10^6^ cells. In consistent with the change of color Doppler imaging, PS and ED blood flow velocity in the RA group with 5×10^5^ and 1×10^6^ cells were significantly improved, but still lower than control.

## Discussion

In the past few years, several studies have revealed that MSCs administration could enhance the recovery of renal injury induced by I/R in experimental models [19], [20]. However, the therapeutic effect of MSCs based therapy is limited by the low efficiency of homing towards clamped kidneys [21]. Although lots of strategies have been shown to increase the strength of homing, the benefits remain modest due to the loss of cell in capillaries of lungs and other organs and short lifetime of the remaining MSCs after systemic administration [13], [15], [16]. Thus, the route of cell delivery may have a crucial role in determining the efficiency of the therapy. Theoretically, the ideal delivery route should be topical so that the transplanted cells can be directly and efficiently homing to the kidney without leakage to other organs, and also not cause extra damage to the host kidney [22].

At present, there are various cell delivery routes for cell therapy in animal models of AKI. As mentioned above, systemic delivery, including intravenous and intra-arterial injections, is the most frequent route for delivery of cells to the kidney after I/R injury. Administration of cells through intravenous route, generally via the tail vein, is much simpler and less invasive than other regular cell delivery routes, but most of injected cells are predominantly trapped in the lungs rather than migrating to the injured kidney [13], [21]. Intra-arterial injection, through the carotid artery, can significantly reduce the trapping of cells in the lung and increase the chance of homing of the injected cells to the kidney. However, the improvement is slight, as the implanted cells widely distribute in various other organs [14]. These findings were also confirmed in our studies. To address the questions pertaining to poor homing of cells after systemic delivery, intraparenchymal injection was used to transplant the cells directly into the renal parenchyma [15], [23]. Despite intraparenchymal injection allowed efficient local delivery of MSCs, the cells tended to gather only at the injection site without being diffused through the kidney. Moreover, intraparenchymal injection after I/R injury might aggravate secondary damage to the kidney and adversely affect the recovery of renal function.

In light of the limitation of the previous methods for cell delivery in injured kidney, we proposed an alternative route to inject MSCs through renal artery, which could combine advantage of both intra-arterial and intraparenchymal injection. Compared with systemic routes, MSCs injection through the renal artery significantly increased homing efficiency to the kidney. After intra-renal artery delivery in the current study, we observed a homogeneous dissemination of the donor cells in the I/R-injured kidney. As kidney is an organ particularly rich in blood vessels, renal artery delivery permits accurate implantation of a larger number of cells and precise targeting of the injured kidney. However, renal artery puncture and injection is highly invasive in rat model of AKI, which can cause massive bleeding and bring even more serious secondary damage than intraparenchymal injection [24]. In this study we developed sub-adventitial renal artery puncture to successfully avoid bleeding from puncture site. Moreover, our results demonstrated that sub-adventitial renal artery puncture and injection would not cause any extra damage to the kidney.

Although renal artery injection was safe and lead to improved cell retention in the kidney, an interesting finding was that the renal function even further deteriorated, after 1×10^6 ^MSCs (the amount commonly used for systemic delivery) were delivered through this route. It has been reported that direct local arterial administration of stem cells increase risk of tissue injury associated with occlusion and embolization [25], . In this study, we assumed that the cells could be trapped in the glomerular capillary loops and stop the blood flow. Doppler color ultrasonography following renal artery injection confirmed an obvious reduction of the blood flow signal in the kidney, when 1×10^6 ^MSCs were applied. Then we decreased cell numbers, and found that 1×10^5 ^MSCs was the optimal dosage for the treatment of AKI through renal artery injection. Our data indicated that 1×10^5 ^MSCs delivered through renal artery was even more effectively than 1×10^6 ^MSCs transplanted through carotid artery or tail vein in improving renal morphology and function after I/R injury. Although the adverse effects associated with cell therapy appear to be limited, there is still an uncertain relationship between the amount of administrated allogeneic MSCs and their long term consequences to the host, and fewer cells are evidently preferred when the outcomes are the same or even better.

In previous studies about cell therapy for kidney repair through renal artery administration, the renal blow flow was blocked by clamping the aorta during the renal artery injection, and 1–3×10^6 ^MSCs were suggested [27]–[29]. Actually, due to the blocked blood flow, most of the injected cells were left in the aorta and renal artery after injection and flushed away when blood flow was restored. Moreover, blocking the renal blood flow during cell injection may further exacerbate renal injury and offset the extra benefit from renal artery injection, especially in the model of I/R injury where the hemodynamics is the main pathogenic factor. In our study, 33 G needle was applied to puncture the renal artery directly to infuse MSCs without blocking the blood flow of the kidney after reperfusion. Through the entire process of cell injection, the blood supply of the kidney was unaffected and the bleeding of punctual site was well controlled.

In summary, administration route and dosage are two critical factors determining the efficiency of cell therapy, and our study showed that 1×10^5^ MSCs injected through renal artery produced the most dramatic improvement in renal function and morphology in rat model of AKI. Renal artery injection allows MSCs to enter the damage kidney more efficiently and homogeneously, however, implanted MSCs localize to glomeruli which may cause occlusion and reduce renal blood flow. Therefore, not too many cells are preferred for locally renal artery delivery.
