# Correction: Maximum Efficacy of Mesenchymal Stem Cells in Rat Model of Renal Ischemia-Reperfusion Injury: Renal Artery Administration with Optimal Numbers

**DOI:** 10.1371/journal.pone.0101336

**Published:** 2014-06-24

**Authors:** The PLOS ONE Staff

The first two authors, Jieru Cai and Xiaofang Yu, should be noted as contributing equally to this work.

The second to last author’s name should be capitalized to read Jie Teng.
